# Imprime PGG, an innate immunomodulator for cancer immunotherapy has the potential to modulate macrophages in the tumor and the spleen to an anti-tumor M1-like phenotype

**DOI:** 10.1186/2051-1426-3-S2-P404

**Published:** 2015-11-04

**Authors:** Kathryn Fraser, Nadine Ottoson, Xiahong Qiu, Annisa SH Chan, Adria Jonas, Takashi Kangas, Jeremy Graff, Nandita Bose

**Affiliations:** 1 Biothera, Eagan, MN, USA

## 

Imprime PGG (Imprime) in combination with anti-VEGF monoclonal antibody, bevacizumab has shown promising clinical efficacy in randomized Phase II clinical trials in non-small cell lung cancer (NSCLC) patients. Imprime, a soluble, yeast β-glucan acts as a pathogen associated molecular pattern (PAMP). As such, Imprime is efficiently and effectively recognized by the cells of innate immune system-macrophages, monocytes and neutrophils- and triggers a coordinated anti-cancer immune attack in concert with other cancer therapies.

In vitro mechanistic studies using whole blood from human volunteers have demonstrated that Imprime may alter the polarization and functionality of monocyte-derived M2 macrophages, reducing surface expression of CD163, upregulating PD-L1, driving CD4 and CD8 T cell expansion and enhancing Th1 polarization. In this study, we sought to evaluate whether Imprime treatment may also similarly affect the polarization state of the macrophages *in vivo*, particularly at a tumor site. Athymic nude mice were injected with the H1299 NSCLC cells. Mice were randomized to treatment groups (n = 10 per group) once tumors reached a group mean of ~100mm^3^. Mice were treated with vehicle, bevacizumab (5 mg/kg twice weekly IP), Imprime (1.2mg/mouse twice weekly IV) or bevacizumab + Imprime. Tumor growth inhibition was calculated at the end of study (% TGI). Tumor and spleen tissue was harvested at the end of study and analyzed by flow cytometry and RT-PCR. CD11b+/CD68+/F4/80+ cells (i.e. murine macrophages) harvested from the spleen of mice treated with Imprime + bevacizumab showed a significant increase in protein expression of inducible nitric oxide synthase (iNOS2) in concert with a significant decrease in Arginase-1 (Arg-1) when compared to the same cells harvested from mice treated only with bevacizumab. Furthermore, *ex vivo* treatment with LPS significantly enhanced the production of TNFα in the CD11B+/CD68+/F4/80+ cells from mice treated with Imprime + bevacizumab. Remarkably, CD11b+ cells harvested from the tumor tissue of these mice showed a similar shift toward an M1-like phenotype, demonstrating significant increases in iNOS2 and PD-L1 expression, and significantly reduced expression of Arg-1, at both the RNA and protein levels. Furthermore, ELISA data from these tumors showed a significantly reduced expression of the potent immunosuppressor, TGFβ, when compared to tumors from mice treated only with bevacizumab, particularly in the most-responsive tumors (> 50% TGI). Collectively, these data show for the first time that Imprime treatment alters the immune microenvironment of a tumor *in situ*, driving a shift to an M1-like polarization state.

